# Vulvar Care: Reviewing Concepts in Daily Hygiene

**DOI:** 10.3390/healthcare13131523

**Published:** 2025-06-26

**Authors:** Jean-Marc Bohbot, Claudio Rebelo, Rossella E. Nappi

**Affiliations:** 1 Department of Genito-Urinary Medicine, Institut Alfred Fournier, 75014 Paris, France; 2 Invicta Saude Clinic, 4200-284 Porto, Portugal; claudio.ginecologia@gmail.com; 3 Department of Pediatrics and Women’s Health, Hospital Pedro Hispano, 4464-513 Matosinhos, Portugal; 4 Department of Clinical, Surgical, Diagnostic and Pediatric Sciences, University of Pavia, 27100 Pavia, Italy; rossella.nappi@unipv.it; 5 Research Center for Reproductive Medicine, Gynecological Endocrinology and Menopause, IRCCS San Matteo Foundation, 27100 Pavia, Italy

**Keywords:** vulva, microbiota, hydration, syndet, soap, detergent, guidelines, microbiology, skin barrier, emulsions

## Abstract

Vulvar hygiene is an important part of general hygiene: the goals are to clear the vulvar area of microbial and cellular debris and vaginal and fecal secretions, ensure local comfort, provide natural levels of hydration, and protect the vulvar microbiota. There are few national and international guidelines on vulvar hygiene. We searched the PubMed database up until 30 November 2024, using logical combinations of the following terms: hygiene, washing, vulva, vulvar, microbiota, hydration, syndet, soap, detergent, water, and customs. The abstracts were reviewed, and potentially relevant full-text articles were retrieved and examined. The subregions of the vulva vary with regard to the presence of sweat and sebaceous glands, the keratin content, the water content, the pH, and the microbiota (notably *Lactobacillus*, *Corynebacterium*, *Staphylococcus*, and *Prevotella*). An alteration in the vulvar microbiota can cause an imbalance in the vaginal microbiota, and vice versa. Vaginal douching may have negative effects on vulvar microbiota. Hair removal might increase the risk of long-term dermatological complications. Repeated washing with water alone exposes the stratum corneum to damage, and washing with soap alters the stratum corneum proteins and lipids, increases skin water loss, and accentuates the risk of irritation. Syndet-based products have a mild detergent effect, promotion of hydration, a suitable pH for the vulvar area, and protection of the vulvar microbiota. Syndet-based products (containing a blend of surfactants, emollients, antioxidants, and buffering agents) appear to be the most appropriate for vulvar care.

## 1. Introduction

For centuries, personal hygiene has been a special part of general hygiene. In fact, personal hygiene is driven not only by a desire for cleanliness but also by social conventions, religious beliefs, and, more recently, the impact of media and advertising. Many researchers have highlighted the influence of personal hygiene on vaginal health and have recommended a few rules with regard to the products to be used. However, environmental movements (such as the “no soap” movement) have recommended banning all chemicals from daily hygiene and using water alone for washing. We therefore considered that it is important to specifically review the anatomical, physiological, microbiological, and environmental aspects of intimate hygiene of the vulvar zone.

## 2. Materials and Methods

In order to inform this narrative review, we searched the PubMed database up until 30 November 2024, using logical combinations of the following keywords: hygiene, washing, vulva, vulvar, microbiota, hydration, syndet, soap, detergent, water, and customs. Publications of all types in English and French were considered. A total of 985 abstracts were reviewed, and 39 relevant full-text articles were retrieved and examined. Furthermore, the reference sections of the selected articles were checked for other relevant publications. Lastly, the authors’ personal libraries were searched for relevant publications.

## 3. Results and Discussion

### 3.1. Physiological and Anatomic Aspects of the Vulva

#### 3.1.1. Anatomy

The vulva extends from the mons pubis at the front to the anus at the back and is limited laterally by the inguinal folds [1]. It has several subregions. Firstly, the vestibule constitutes the border between the vulva and vagina. It includes (from front to back) the glans of the clitoris, the urinary meatus (flanked by the Skene’s gland openings), and the vaginal orifice. Secondly, the labia minora are hairless and moist; the dermis contains sweat glands and many sebaceous glands, which secrete vulvar smegma. The labia minora are among the erogenous zones. Lastly, the labia majora are separated from the labia minora by the interlabial sulcus. These labia are hairy and contain many sebaceous and sweat glands. The labia majora are also among the erogenous zones. Bartholin’s glands (also known as the greater vestibular glands) are located at either side of the external orifice of the vagina. Their secretions have a major role in producing lubrication during sexual intercourse. The clitoris is a complex organ formed by the corpora cavernosa (also known as the clitoral roots, which merge forward to form the body of the clitoris—the visible portion of which is the glans) and the vestibular bulbs that surround the vaginal orifice and join the corpora cavernosa [2].

Histologically, the vulva is covered by a keratinized epithelium that gradually decreases in thickness from the mons pubis to the inner side of the labia minora, where it becomes very thin. The vestibule is covered by a non-keratinized epithelium, which is structurally similar to that of the vagina [3].

#### 3.1.2. Vulvar Hydration

The literature data on the water content of the vulvar skin are contradictory. According to Fujimura et al., the water content of the vulva (including the mons pubis) is lower than that of other skin sites, such as the forearm and groin [4]. In contrast, another study found that the vulvar area’s water content was similar to that of other skin sites or was even slightly higher [5]. Furthermore, Fujimura et al. reported a significant difference between water loss in the vulvar region vs. the forearm and thigh, which suggested that the skin’s barrier effect was more limited in the vulvar area [4]. However, this barrier effect is essential for good local skin health, due to repeated exposure of the area to irritants such as urine, vaginal secretions, and feces. Given the contradictory nature of the literature data on the vulvar skin’s water content, well-designed comparative studies using standardized methodologies (e.g., corneometry and transepidermal water loss assessments) could be usefully conducted. These studies should consider variations due to age, hormonal status, and ethnicity, in order to clearly delineate normative hydration values.

#### 3.1.3. Vulvar Microbiota

Although the composition of the vaginal microbiota has been well characterized (i.e., lactobacilli dominance in non-menopausal women), the vulvar microbiota has been studied in much less detail but constitutes a “crossroads” between the cutaneous, anorectal, vaginal and urinary microbiotas. Furthermore, the vulvar microbiota appears to vary markedly from one woman to another and is not dominated by a particular microbial species.

A meta-analysis of 10 studies in healthy women reported the presence of *Lactobacillus*, *Corynebacterium*, *Staphylococcus* and *Prevotella* [6]. However, the exact composition of the vulvar microbiota depends on the anatomical site (i.e., the mons pubis, the labia majora, the labia minora, the vestibule, etc.). The composition of the vulvar microbiota does not appear to vary over the course of the menstrual cycle. In contrast, one study highlighted a significant difference in vulvar microbiota composition between thin women (with a body mass index between 18 and 25) and obese women (with a body mass index > 30) [7]. Relative to thin women, the obese women had a lower prevalence of *Lactobacillus* spp. and a higher prevalence of *Corynebacterium* spp. and *Anaerococcus* spp.

The *Lactobacillus* found in the vulva can come from the vagina or the rectum. Indeed, the rectum is known to be a natural reservoir of certain strains of vaginal lactobacilli, which cross the vulvoperineal area to the vestibule and the vagina. During this transit, lactobacilli are vulnerable to the antiseptic compounds contained in certain hygiene products, the inhibitory action of which has been evidenced in vitro [8].

Better characterization of the vulvar microbiota through comprehensive longitudinal studies of diverse populations is crucial. The use of advanced molecular sequencing technologies, including metagenomics and transcriptomics, could provide deeper insights into vulvar microbial communities, their stability, and responses to external hygiene practices.

#### 3.1.4. Vulvar Dysbiosis and Genital Disorders

The microbial dialogue between the vulva and the vagina is bidirectional: on one hand, the vestibular microbiota is markedly influenced by the vaginal microbiota; on the other, the vulva is an transition area in which cutaneous and intestinal micro-organisms move towards the vagina [6,9,10]. Thus, an alteration in the vulvar microbiota can lead to an imbalance in the vaginal microbiota, potentially resulting in conditions such as BV; this is characterized by an alteration in which *Lactobacillus* spp. diminishes and other anaerobic species (such as *Gardnerella* spp., *Prevotella* spp., and *Mobiluncus* spp.) dominate.

In some studies, changes in vestibular microbiota have been correlated with vestibulitis and vulvodynia [11,12]. The presence of *Lactobacillus gasseri*, BV-associated bacteria (*Gardnerella*, *Ureaplasma*, *Mycoplasma*, etc.) and/or *Bifidobacterium* appeared to be linked to the presence of these clinical conditions [12].

Thus, vulvar dysbiosis can be associated with purely vulvar disorders but also with vulvovaginal disorders.

#### 3.1.5. Vulvar pH

The skin’s pH varies from site to site but is typically between 4 and 6 [13]. Folds and moist areas like the vulva have a higher pH (around 6) than dry areas [13]. The vulvar pH also depends on the site—particularly in the vestibule, where the pH is close to that of the vagina (about 4.5). Thus, the area exposed to personal hygiene procedures and products does not have a uniform, physiological pH value; the gradient runs from 4.5 in the vestibule to 6 in the surrounding skin area. Vulvar pH also varies as a function of exogenous factors, such as contact with vaginal secretions, menstruation, urine, feces, and age.

### 3.2. Personal Hygiene

An international study of about 10,000 women from 10 countries found that the proportion performing intimate care daily varied greatly from one country to another (from 38% to 91%) [14]. The study also found that talking about the intimate area of the body is taboo for many women and notably among younger women. In another international survey of 4246 postmenopausal women aged 55 to 65, 60% of the respondents considered that embarrassment prevents many women from talking to their healthcare provider about menopause-related vaginal discomfort [15].

The goals of personal intimate hygiene are to clear the vulvar area of microbial and cellular debris, remove vaginal and fecal secretions that may have settled on the area, and ensure local comfort. Washing should not alter the stratum corneum, the surface hydrolipid film, the pH, or the skin microbiota. For optimal effectiveness, personal intimate hygiene must therefore achieve two fundamental objectives: natural levels of hydration and preservation of the natural vulvar microbiota. Indeed, water is lost more readily from the vulva than from nearby tissues [4]; hence, the use of inappropriate products exposes the vulvar area to a risk of dehydration. Furthermore, vulvar dysbiosis increases the likelihood of local complications (e.g., irritation, inflammation, and vulvar infections) and vaginal dysbiosis, since the vulvar and vaginal microbiota are connected [16,17].

### 3.3. Country-Specific Habits and Practices

Personal hygiene is highly dependent on local customs, ethnicity, or religious principles, and so a variety of behaviors are observed in different parts of the world [14,18]. Various “dry sex” vaginal insertions such as herbs, rock dust, cotton, cloths or moisture-absorbing paper are still performed in a few countries, as well as vaginal injections of vinegar, lemon juice, salt water, or other detergents. This practice exposes the vagina to mucosal damage but may not necessarily be a risk factor for disease, and the impact on vulvar health is not clear [19,20].

### 3.4. Washing with Water

Repeated washing with (or prolonged exposure to) water alone exposes the stratum corneum to damage, which decreases the skin’s barrier effect and thereby increases water loss and skin dryness. Furthermore, one study found that water alone was less effective than a non-antibacterial hygiene product at ridding the skin of bacteria [21].

### 3.5. Soap vs. Synthetic Detergents (Syndets)

Traditionally, soap is a salt mixture of animal- or plant-derived fatty acids and sodium hydroxide, formed by saponification. Syndets are combinations of petroleum-derived or oil-based washing agents. Conventional soap generally has a pH of between 8.5 and 11, while syndet has a pH of between 5.5 and 7 (i.e., closer to the skin’s pH of between 4 and 6). Successive washes with conventional soap alter the stratum corneum proteins and lipids and increase skin water loss, with risk of irritation and dryness [22,23]. In contrast, the use of syndet does not alter the stratum corneum proteins and lipids [23]. Given that the vulvar region is naturally more exposed to water loss, the use of a syndet-based intimate hygiene product is more appropriate. The guidelines on female genital hygiene issued by a group of physicians from the Middle East and Central Asia recommended daily washing with a mild detergent (pH 4.2 to 5.6) [24]. Broadly, the syndet-based approach is in line with the European dermatological guidelines on (i) topical care for atopic dermatitis (which cover emollients and non-irritant, low-allergen formulations of cleansing agents such as syndets), (ii) infant skin care (the use of products that are formulated to maintain the skin surface at approximately pH 5.5), and (iii) the management of vulval conditions (the avoidance of contact with soap and shampoo, and the use of emollients) [25,26,27,28].

### 3.6. Antiseptics and Drugs

In 2016, the United States Food and Drug Administration (US-FDA) recommended not using hygiene products containing certain antiseptics, such as triclosan or triclocarban [29]. Clinical studies have confirmed that antibacterial hygiene products have a negative impact on both the integumentary ecosystem [8,30] and the environment. One study showed that the use of antiseptic products for vulvar (and/or vaginal) washing increased the risk of BV by a factor of three [31].

Vulvar involvement in adverse drug reactions has also been reported. The glucosuria induced by treatment with sodium-glucose co-transporter receptor-2 inhibitors has been linked to recurrent vulvovaginal candidiasis and vulvar skin damage [32].

### 3.7. Vaginal Douching

Since the 1980s, a large body of evidence has shown that vaginal douching poses a danger to the microbiota and vaginal mucosa. Indeed, regular vaginal douching has been associated with an elevated risk of BV, sexually transmitted infections, and upper genito-urinary tract infections [33]. In patients using a lactic acid vaginal douche three times a week, the prevalence of *Candida albicans* increased—despite the hygiene product’s acidic pH of 3.5 [34]. Despite being informed about the harmful effects of vaginal douching, many women continue to perform this procedure: according to various studies, 29% to 92% women had douched at least once in the preceding 3 months [35,36]. These women considered that vaginal douching improves vaginal hygiene (especially after sexual intercourse), avoids infections, and limits vaginal odors.

### 3.8. What Are the Main National and International Guidelines on Personal Intimate Hygiene?

Information and educational campaigns on hygiene during menstruation have been conducted in various countries, with a view to promoting best practice during this particular period. In contrast, there are few official national and international guidelines on personal hygiene in general. Although the 2013 guideline from the United Kingdom’s Royal College of Obstetricians and Gynecologists (now integrated into the UK National Guideline on the Management of Vulval Conditions, issued by the British Association for Sexual Health and HIV) provides advice on hygiene (recommending the use of ointments and the avoidance of soap), the focus is on patients with vulvar disorders [37]. Similar advice was given in the 2016 European guidelines [38]. As mentioned above, the guidelines on female genital hygiene issued by a group of physicians from the Middle East and Central Asia recommended daily washing with a mild detergent [24]. Lastly, the European guidelines on the management of vulval conditions recommend the avoidance of contact with soap and shampoo, and the use of emollients [28].

The general principles for daily personal hygiene are as follows: (i) avoid washing with water alone (due to the risk of dryness or irritation with prolonged or repeated exposure to water); (ii) avoid conventional soaps, shower gels, and bath foams; (iii) avoid antiseptic products; (iv) avoid vaginal douching; (v) use liquid syndets and rinse thoroughly; (vi) avoid washcloths (use the hands instead); and (vii) wash once or twice a day [39,40,41].

### 3.9. Special Cases

#### 3.9.1. Menstruation

During menstruation, a morning wash and an evening wash are recommended. Washing can also be recommended after tampon or cup changes. The instructions for correct menstrual tampon use must be strictly followed, in order to avoid toxic shock syndrome (TSS, a systemic infection caused by the contamination of menstrual blood with strains of toxin-producing *Staphylococcus aureus*). The use of menstrual cups does not eliminate the risk of TSS [42,43]. Experts have also emphasized the value of thorough handwashing prior to placement of the tampon or cup. Sanitary pads and other external protections do not expose users to a risk of TSS according to a survey conducted by France’s Agence nationale de sécurité sanitaire de l’alimentation, de l’environnement et du travail (ANSES, the Agency for Food, Environmental and Occupational Health and Safety) [44]. Furthermore, a committee of experts from the ANSES [44] has recommended better documentation of the composition of menstruation products because traces of pesticides (lindane, glyphosate, etc.), phthalates, volatile organic compounds, and polycyclic aromatic hydrocarbons have been detected in tampons or sanitary protection products. The committee also recommended the removal of all perfumes from these devices. Similarly, elevated levels of 1,4-dichlorobenzene were observed in regular users of vaginal douches.

#### 3.9.2. Intimate Area Hair Removal

This practice is becoming increasingly common among men and women. One study showed that 84% of American women had removed hair from the pubis alone or from the entire anogenital area [45]. Depending on the type of intimate hair removal, individuals are exposed to various complications: superficial wounds, burns, and erythema. Pruritus and scratching lesions can be observed during hair regrowth after removal. The relationship between intimate hair removal and the incidence of sexually transmitted diseases is subject to debate. Since genital hair is a natural physical barrier to vulvar environmental stressors, hair removal might expose the person to long-term complications—especially if the entire hair follicle hair is permanently removed [41]. Further characterization of these risks is required.

#### 3.9.3. Sexual Intercourse

Personal hygiene is usually recommended before sexual intercourse. However, the role of olfactory stimulation in sexual arousal may alter this behavior. Famously, Napoleon is said to have written to Josephine: “Don’t wash, I’ll hurry and be home in a week”.

Vulvovaginal and axillary secretions often contribute to male sexual arousal. One study showed that male olfactive exposure to pre-ovulation axillary odors and (especially) vulvar odors was associated with higher saliva levels of testosterone and cortisone [46]. In any case, the incorporation of deodorants or perfumes in personal hygiene products used before sexual intercourse is not recommended [37,38].

Some women perform personal hygiene procedures immediately after sexual intercourse—mainly to avoid pregnancy and/or infections. Firstly, personal hygiene is not an effective method of contraception, and secondly, it does not prevent sexually transmitted infections (STIs). Women with BV complain of a bad odor after sexual intercourse, which prompts them to wash the vulva and (often) douche the vagina. However, it is now clear that vaginal douching worsens vaginal dysbiosis [33].

#### 3.9.4. The Menopause

The menopause and the associated estrogen deficiency have a significant impact on genital health. The signs and symptoms are referred to generically as genito-urinary syndrome of menopause (GSM), with vulvovaginal dryness, irritation, burning, dyspareunia, urinary incontinence, recurrent urinary tract infections, abnormal leukorrhea, etc. This very common syndrome (affecting 36% to 90% of women, depending on the study) also affects the individual’s quality of life.

After the menopause and in the absence of hormone replacement therapy, the vulvar epithelium becomes thinner and sometimes paler, erythematous, and progressively atrophic. Conventional treatments include topical estrogens and/or moisturizing products. In the latter group, hyaluronic acid has demonstrated efficacy [47] and safety—especially in women with a history of breast cancer [47,48].

In postmenopausal women, reduced estrogen levels lead to significant physiological changes in vulvar and vaginal tissues; these changes result in greater epithelial fragility, lower hydration, and heightened susceptibility to irritation and contact dermatitis [49]. Frequently used over-the-counter products (including talcum powders, topical anesthetics containing benzocaine, and various barrier ointments (e.g., petroleum-based or zinc oxide preparations)) have been shown to exacerbate vulvovaginal irritation or provoke adverse dermatological responses in postmenopausal women. Erekson et al.’s study indicated that a substantial proportion of postmenopausal women regularly apply several of these products simultaneously, which intensifies their vulnerability to dermatologic complications [49]. Clinical guidance on postmenopausal vulvar hygiene should therefore emphasize the need to avoid products with irritative or allergenic potential and advocate mild, hypoallergenic, fragrance-free, and pH-balanced formulations specifically designed to preserve epithelial integrity, maintain moisture balance, and minimize microbial dysbiosis and irritative sequelae.

These products can be applied vaginally but also to the vulva. Personal hygiene can also improve levels of comfort for women with GSM; a syndet with moisturizing and soothing components (glycerin or herbal extracts, for example) can decrease the feeling of dryness. In women with urinary and/or fecal incontinence, the use of wipes soaked in cleansing products (liquid syndet) and moisturizing agents has been shown to protect the skin more effectively than conventional soap [50]. However, the small size of these studies limits their robustness and emphasizes the need for larger studies. The symptoms of GSM make it even more important for postmenopausal women to avoid unsuitable cleansing products and hygiene practices.

### 3.10. Environmental Impact

To the best of our knowledge, the environmental impact of personal hygiene procedures and products has not been specifically assessed. The use of menstrual products has been highlighted as a source of potentially irritating environmental chemicals, although the level of exposure and the impact on health are subject to debate [51,52,53].

However, some data on hand washing are available. Hand washing with solid soap uses 2–4 L of water and generates 15–18% of a household’s wastewater; the subsequent spreading of surfactants can adversely affect aquatic flora and fauna [54,55]. As mentioned above, antiseptic soap is of no value in personal intimate hygiene. It also has a greater environmental impact than conventional soap. Biodegradable, plant-derived washing products (green chemistry) with environmentally friendly production methods (e.g., fermentation) are now being designed and developed [54]. Lastly, the European authorities are encouraging manufacturers to reduce the weight of packaging and to favor renewable or biodegradable packaging.

At present, environmental considerations have led solely to guidelines on using only small amounts of hygiene products and small volumes of water (i.e., not letting the tap run while washing).

### 3.11. The Choice of a Product for Personal Intimate Hygiene

Some experts consider that hygiene products for the vulvar area should have the following characteristics: (i) hypoallergenic and soap-free, with a mild “detergent” effect; (ii) no irritants, and good tolerability; (iii) promotion of hydration, and protection against dryness; (iv) maintenance of a balanced microbiome; (v) a blend of surfactants, emollients, and buffering agents, with a suitable pH for the vulvar area [56].

Research summarized in a Master’s thesis published in 2013 compared the five most widely sold intimate hygiene products on the Portuguese market with solid (bar) soap, in terms of the impact on three epidermal variables: the pH, the lipid barrier, and the level of hydration [57]. The hygiene products were divided into three main groups, according to the main surfactants present: an amphoteric surfactant (both positively and negatively charged), an anionic surfactant (negatively charged), or a mixture of amphoteric and anionic surfactants. The effects of the products (evaluated 30, 60 and 120 min after application) varied with the type of surfactant present. Use of the bar soap was associated with the highest (most alkaline) pH values up to 4 h after application. With regard to the lipid barrier, the bar soap had a stronger detergent action than the hygiene products. It removed the hydrophilic layer totally and thus promoted late and persistent dehydration. The five hygiene products all had similar behaviors with regard to pH: a physiological pH value was restored 1 h after application. The product containing anionic surfactants showed a more pronounced delipidating action and more dehydration, whereas the four other products did not have a major impact on skin lipid removal.

A product containing amphoteric surfactants gave the best hydration values for vulvar skin (i.e., minimal reduction in hydration in the labia majora area). A greater reduction in hydration was observed with the product with amphoteric + anionic surfactants, due to the presence of sodium lauryl sulphate. Anionic surfactants were more irritating still, with alterations in the stratum corneum function and thus greater vulnerability to injury or trauma.

The researchers concluded that given its strong alkalinity and lipid-depleting power, bar soap is certainly the wrong product for female intimate hygiene. Although all the other products could be used for intimate hygiene, the ones without anionic surfactants had a weaker delipidating action and better hydration.

A study conducted in 2017 found that the appropriate selection of vulvar hygiene products is crucial for intimate health [40]. The external vulvar region is sensitive to irritation and has unique physiological and microbiological properties. Harsh soaps and detergents may disrupt its natural pH and microbiota, potentially causing irritation or infections. The clinical guidelines recommend using gentle, hypoallergenic cleansers with mild detergency and a balanced pH [39]. Specifically formulated intimate washes may help maintain microbiota balance, support skin health, and reduce the risk of infections. Regular, gentle cleansing with appropriately formulated products is considered beneficial for overall intimate hygiene and vulvar health, particularly as adjunct care for the prevention of recurring conditions such as BV.

Despite growing interest in vulvar hygiene, many existing studies in this field are limited by small sample sizes and insufficient statistical power, which hinder the generalizability and reliability of the findings. Future studies of vulvar care should focus on larger sample sizes, in order to enhance statistical power and generalizability. Multicenter collaborations would be valuable for overcoming the limitations of previous small-scale studies and facilitating robust data collection.

### 3.12. Natural Extracts, Safety and Tolerability

The value of adding natural plant extracts to intimate hygiene products has been studied extensively. In a 2008 article, 2641 women were asked to use intimate hygiene products containing natural plant extracts for 4 weeks [56]. A positive pre-post clinical effect was observed, with a reduction in vaginal pH and symptoms and an increase in the quality of sexual activity.

In a double-blind, controlled study published in 2020, 40 women were randomized to the twice-daily use of a syndet based on natural extracts or one based on lactic acid [58]. Although both cleansing products performed well in terms of safety and tolerability, the natural extract-based product was better tolerated and more effective for some variables (less dehydration and a higher sebum content in the labia majora and minora) than the lactic acid-based product.

## 4. Conclusions

Personal intimate hygiene has an important role in women’s genital health. Advice on good hygiene practices should be an integral part of the counselling given by healthcare professionals. This is especially important for younger women because the vulvovaginal health information on social media platforms has extensive shortcomings [59].

Although the recommendations on vulvar hygiene depend on the stage of the woman’s life, a number of principles are always applicable: (i) avoid washing with conventional soaps, antiseptic products, or water alone, (ii) use a liquid syndet (preferably one with moisturizing and soothing components), (iii) wash the vulvar area from front to back, (iv) wash once a day, preferably just after bowel voiding, and (v) avoid vaginal douches.

Taken as a whole, the choice of a cleansing product should be guided by its locally hydrating properties (e.g., liquid syndets with moisturizing and soothing components), its ability to preserve the vulvar microbiota, and its appropriateness for the woman’s genital health life stage (menopause, infections, etc.). Last but not least, intimate hygiene practices and products must be environmentally friendly, in order to reduce their impact.

## Data Availability

No new data were created or analyzed in this study.
